# Supplementary material for: Collaborative intelligence in AI: Evaluating the performance of a council of AIs on the USMLE
Source: PLOS Digit Health. 2025 Oct 9;4(10):e0000787. doi: 10.1371/journal.pdig.0000787 (PMC12510544; doi:10.1371/journal.pdig.0000787)
Supplement: S2 File — (ZIP) [file pdig.0000787.s002.zip › S2 Data Raw data files containing unprocessed question inputs and LLM outputs including transcripts of deliberations/STEP3_Question-117-1_Type-MC-NJ.docx]

**Facilitator** **[Round 1]:** A 54-year-old woman, gravida 2, para 2, comes to the office because of a 4-month history of hot flushes that awaken her from sleep on most nights. She also reports increased irritability during this time. She has not had fever, shortness of breath, or cough. She says her appetite has been good and her weight has been stable. Medical history is remarkable for hysterectomy done 5 years ago for symptomatic leiomyomata uteri. The patient takes calcium and vitamin D supplements. She has never smoked cigarettes. She drinks one glass of wine 1 night weekly. She is 165 cm (5 ft 5 in) tall and weighs 48 kg (106 lb); BMI is 18 kg/m². Vital signs are temperature 37.0°C (98.6°F), pulse 66/min, respirations 12/min, and blood pressure 108/64 mm Hg. Physical examination discloses no abnormalities. Screening PPD skin test shows 15 mm of induration. Chest x-ray shows densities consistent with granulomata. Which of the following pharmacotherapies is most likely to alleviate this patient's symptoms? (A) Clonidine (B) Conjugated estrogen (C) Fluoxetine (D) Isoniazid and rifampin (E) Raloxifene

**AI_C:** (1) RESTATING THE QUESTION: An adult woman with a history of hysterectomy is presenting with symptoms of hot flashes and irritability, which have been present for the past four months. Her general health appears to be stable and she has no other symptoms. Additionally, a PPD skin test and chest x-ray indicate tuberculosis, despite the absence of correlating symptoms. We are required to select a course of pharmacotherapy that will be best suited to alleviate her primary symptoms. (2) PLANNING: The current symptoms, notably, the hot flashes and irritability, could be due to various reasons. Considering her clean medical history and lack of symptoms typically associated with heart disease, respiratory disease or cancers, it's reasonable to think that she is experiencing menopausal symptoms. The presence of tuberculosis, indicated by the PPD skin test and chest x-ray, could be an incidental finding as she lacks respiratory symptoms. The list of medications provided as options constitutes treatments for menopausal symptoms and tuberculosis. (3) CLINICAL REASONING: Hot flashes and irritability are common symptoms of menopause. Given that her hysterectomy was performed five years ago, it is plausible that the ovaries stopped functioning effectively sometime afterwards, leading to a drop in estrogen levels and subsequently, the onset of menopause symptoms. Conjugated estrogen would effectively replace this lack of natural estrogen in her body, which could subsequently alleviate the primary symptoms. Despite having granulomas, she exhibits no symptoms related to tuberculosis such as fever, cough, or weight loss and hence, starting her on tuberculosis treatment before further investigation could be extreme. (4) CONCLUDING: Given the aforementioned reasoning process, the woman is most likely experiencing menopausal symptoms due to hypoestrogenism. Appropriate supplemental therapy would aim to replace the deficient estrogen likely causing her symptoms. (5) RESPONSE: Thus, the most appropriate pharmacotherapy to alleviate this patient's symptoms would be (B) Conjugated estrogen.

**AI_B:** (1) RESTATING THE QUESTION: The inquiry pertains to the best pharmaceutical option for a 54-year old woman who has been having sleep interrupting hot flushes and increased irritability for the past 4 months. She has a history of hysterectomy performed 5 years ago due to leiomyoma uteri. Despite having granulomata observed in her chest x-ray, her history and physical examination do not suggest any infectious or systemic diseases. The pharmacotherapies proposed are clonidine, conjugated estrogen, fluoxetine, isoniazid and rifampin, and raloxifene. (2) PLANNING: The primary assumptions in this case are that her hot flashes and irritability are likely due to the physiological changes of menopause and that her prior hysterectomy may have advanced these symptoms by removing the primary source of estrogen production. An alternate assumption could be that her symptoms are attributable to some other underlying medical condition; however, the described physical examination and negative fever suggest otherwise. As such, it would be clinically appropriate to prioritize the assumption that her symptoms are related to menopause. (3) CLINICAL REASONING: Menopause is a physiological process in women typically occurring in their late 40s to early 50s, characterized by the cessation of menstruation due to declining ovarian function and subsequently decreased estrogen levels. Symptoms can vary but often include hot flushes, sleep disturbances, mood alterations, and irritability, as observed in this patient. Given her age and the absence of menstrual cycles following her hysterectomy, it could be inferred that her presenting symptoms are likely due to menopause. Hormone replacement therapy (HRT), including estrogen, is often used to ameliorate these symptoms. (4) CONCLUDING: The given details about the patient: her age, symptomatology, and surgical history, point towards the diagnosis of menopausal symptoms. The goal of therapy in this case would be to ameliorate her symptoms by replacing the missing estrogen that naturally declined as a result of menopause. Estrogen would specifically target and alleviate her hot flushes and mood changes. (5) RESPONSE: Therefore, the most suitable pharmaceutical option to alleviate this patient's symptoms would likely be (B) Conjugated estrogen.

**AI_A:** RESTATING THE QUESTION: We're looking at a case of a 54-year-old woman, having had 2 pregnancies and 2 deliveries previously, is struggling with persistent hot flashes and increased levels of irritability. She appears otherwise healthy, demonstrating no signs of respiratory distress or infection, and maintaining a steady weight. She underwent a hysterectomy half a decade ago due to symptomatic leiomyomata uteri (benign tumors in the uterus), has a clean bill of health apart from some granuloma identified through a PPD skin test and chest x-ray and maintains an appropriate BMI for her height and weight. We're being asked to identify the best pharmacotherapy route to mitigate her said symptoms of hot flashes and irritability. PLANNING: The presence of hot flashes and increased irritability in a post-menopausal woman who had had a hysterectomy, in the absence of other identified causes, suggests the possibility of Hormone Replacement Therapy (HRT) as a potential answer due to estrogen deficiency. Some of the alternatives listed in the question: Clonidine, Fluoxetine, Isoniazid and rifampin, and Raloxifene have different functions. Clonidine is commonly used to manage hypertension and ADHD. Fluoxetine is an SSRI, used in treating mood disorders such as depression and anxiety. Isoniazid and Rifampin are anti-tubercular drugs. Raloxifene is a selective estrogen receptor modulator, used in postmenopausal women but primarily for the prevention of postmenopausal osteoporosis and breast cancer. CLINICAL REASONING: Given that the woman is struggling with symptoms potentially relating to estrogen deficiency, the most judicious course of action would not be to treat for unrelated diseases or conditions, such as hypertension (Clonidine), mood disorders (Fluoxetine), tuberculosis (Isoniazid and Rifampin) or to prevent osteoporosis and breast cancer (Raloxifene), because those scenarios don't fit our patient's clinical picture. In this case, my assumption is that hormone replacement therapy would be the best fitting solution. CONCLUDING: Since the clinical picture points towards a hormonal imbalance likely due to the hysterectomy she’s undergone 5 years ago and she's now presenting with the described symptoms that are characteristic of menopausal syndrome, hormone replacement therapy beckons as the preferable route to take. RESPONSE: Therefore, given her symptoms and medical history, Conjugated estrogen (Option B) would most likely be the best pharmacotherapy to alleviate this patient's menopausal symptoms.

**AI_E:** (1) RESTATING THE QUESTION: We are asked to identify the most appropriate pharmacotherapy for a 54-year-old woman who's been complaining of hot flushes and irritability for the past 4 months. Her medical history includes a hysterectomy for leiomyomata uteri. Her vital signs and physical examination are within normal limits while her chest x-ray indicates densities aligned with granulomata. Gravida 2 and para 2 pertain to a woman's obstetrical history, indicating that she has been pregnant twice and has given birth to two children. Leiomyomata uteri, also known as uterine fibroids, are benign growths in the uterus that may cause symptoms like urinary frequency, constipation, back or leg pain, and in some cases problematic bleeding. (2) PLANNING: The primary symptoms are hot flushes and irritability, commonly associated with menopause which correlates with the patient's age. Given her past hysterectomy, the usual common sign of menopause (cessation of menses) would not be applicable. We can also consider systemic manifestations of tuberculosis due to the granulomatous lesions on her chest x-ray, however, her lack of related symptoms (fever, cough, shortness of breath) makes this less likely. So, contemplated assumptions include menopausal syndromic changes, side effects of any medication she's currently taking, and systemic manifestations of her existing granulomata. Considering her age and the fit with her symptomology, menopause is the most clinically relevant hypothesis to pursue. (3) CLINICAL REASONING: Once a woman reaches the age category of this patient and starts experiencing such symptoms, menopause becomes likely. The presence of night sweats, hot flushes, and mood changes like irritability or mood swings are typical presentations. This is attributed to a decline in the levels of estrogen and progesterone. (4) CONCLUDING: Following the rationale of menopausal symptoms, the clinician's aim is to mitigate these symptoms and improve the patient's quality of life. Hormone replacement therapies can mimic the functions of the diminishing hormones, such as conjugated estrogens. (5) RESPONSE: Given this analysis, I infer that (B) Conjugated estrogen is most likely to alleviate this patient's symptoms. The estrogen therapy can effectively reduce her menopausal symptoms. Other options on the list don't directly address menopausal symptoms or are unrelated to her condition. For example, isoniazid and rifampin (option D) would be appropriate for someone diagnosed with tuberculosis, which she does not seem to have.

**AI_D:** RESTATING THE QUESTION: The question appears to seek information regarding the most effective pharmacotherapy for a post-menopausal woman presenting with hot flashes and increased irritability. 'Gravida 2, para 2' refers to the lady having had two pregnancies and two deliveries. The term 'leiomyomata uteri' is the medical term for fibroids in the uterus, which led to the woman undergoing a hysterectomy, the surgical removal of the uterus. A PPD test showing 15mm induration indicates possible tuberculosis infection, and densities viewed on a chest x-ray are results consistent with granulomata, a form of inflammation often related to infectious diseases. PLANNING: The assumptions in this case involve the woman's symptoms being related to the post-menopause phase (hot flashes and irritability), or alternatively, her symptoms could be a result of the possible tuberculosis infection. While tuberculosis primarily presents with symptoms like cough, fever, and weight loss, it can occasionally manifest in non-specific symptoms. Further analysis, the patient's presentation more accurately mirrors post-menopausal symptoms, particularly given her age and hysterectomy history. CLINICAL REASONING: Addressing these symptoms with parallel treatment for her possible tuberculosis might be an approach, however, we can't assume the symptoms are due to TB infection because she doesn't present the common TB symptoms. Pharmacotherapy related to menopausal symptoms includes hormone replacement therapy (HRT), antidepressants, or other options. Conjugated estrogen (option B) is a form of HRT often used in managing menopausal symptoms like hot flashes, while fluoxetine (option C) is an antidepressant that can sometimes be used in treating irritability associated with menopause. Clonidine (option A) is primarily used as an antihypertensive but can occasionally help with hot flashes. Raloxifene (option E) is a selective estrogen receptor modulator often used in the prevention of osteoporosis in post-menopausal women, though it is not a primary treatment for menopausal symptoms. Isoniazid and rifampin (option D) are medications used in the treatment of tuberculosis, which may not specifically alleviate the patient's presenting symptoms. CONCLUDING: Therefore, to prioritize the most distressing symptoms which are the hot flushes and increased irritability, which could be utilizing a pharmacotherapy that addresses these symptoms directly in postmenopausal women, the option seems to be the one that best serves this need. RESPONSE: Given the patient's symptoms of hot flashes and irritability post-menopause, and considering the drugs listed, (B) Conjugated estrogen stands out as the most likely pharmacotherapy to provide her symptomatic relief.

**Facilitator** The consensus among the AI responses is that the most appropriate pharmacotherapy for the 54-year-old woman presenting with hot flashes and increased irritability is Conjugated Estrogen (Option B). This conclusion is based on the assumption that her symptoms are likely due to menopause, which is consistent with her age and history of hysterectomy. The hot flashes and irritability are common symptoms of menopause, often resulting from a decline in estrogen levels. Conjugated estrogen, a form of Hormone Replacement Therapy (HRT), can effectively replace the deficient estrogen in her body, potentially alleviating her symptoms. Other options listed, such as Clonidine, Fluoxetine, Isoniazid and Rifampin, and Raloxifene, are primarily used for conditions that do not align with the patient's current clinical picture. Therefore, the consensus is that Conjugated Estrogen is the most suitable pharmacotherapy to alleviate this patient's menopausal symptoms.
